# Overview of Native Chicken Breeds in Italy: Small Scale Production and Marketing

**DOI:** 10.3390/ani11030629

**Published:** 2021-02-27

**Authors:** Alessandro Franzoni, Marta Gariglio, Annelisse Castillo, Dominga Soglia, Stefano Sartore, Arianna Buccioni, Federica Mannelli, Martino Cassandro, Filippo Cendron, Cesare Castellini, Alice Cartoni Mancinelli, Silvia Cerolini, Ahmad Abdel Sayed, Nicolaia Iaffaldano, Michele Di Iorio, Margherita Marzoni, Sonia Salvucci, Achille Schiavone

**Affiliations:** 1 Dipartimento di Scienze Veterinarie, Università degli Studi di Torino, Largo Paolo Braccini 2, 10095 Grugliasco, Italy; alessandro.franzoni@unito.it (A.F.); marta.gariglio@unito.it (M.G.); annelisse.castillogarrido@unito.it (A.C.); dominga.soglia@unito.it (D.S.); stefano.sartore@unito.it (S.S.); 2 Dipartimento di Scienze e Tecnologie Agrarie, Alimentari, Ambientali e Forestali, Università di Firenze, Via delle Cascine 5, 50144 Firenze, Italy; arianna.buccioni@unifi.it (A.B.); federica.mannelli@unifi.it (F.M.); 3 Department of Agronomy, Food, Natural Resources, Animals and Environment (DAFNAE), Università di Padova, Viale dell’Università 16, 35020 Legnaro, Italy; martino.cassandro@unipd.it (M.C.); filippo.cendron@phd.unipd.it (F.C.); 4 Dipartimento di Scienze Agrarie, Alimentari e Ambientali, Università di Perugia, Borgo XX Giugno 74, 06121 Perugia, Italy; cesare.castellini@unipg.it (C.C.); alice.cartonimancinelli@unipg.it (A.C.M.); 5 Dipartimento di Medicina Veterinaria, Università degli Studi di Milano, Via dell’Università 6, 26900 Lodi, Italy; silvia.cerolini@unimi.it (S.C.); ahmad.abdel@unimi.it (A.A.S.); 6 Dipartimento Agricoltura, Ambiente e Alimenti, Università degli Studi del Molise, Via Francesco De Sanctis, 86100 Campobasso, Italy; nicolaia@unimol.it (N.I.); michele.diiorio@unimol.it (M.D.I.); 7 Dipartimento di Scienze Veterinarie, Università di Pisa, Viale delle Piagge 2, 56124 Pisa, Italy; margherita.marzoni@unipi.it (M.M.); sonia.salvucci@unipi.it (S.S.)

**Keywords:** Italian poultry breeds, avian biodiversity, autochthonous poultry, small-scale production, products market

## Abstract

**Simple Summary:**

The loss of biodiversity is a matter of great concern worldwide. In the agricultural sector, the industrialization of livestock farming and the wide-spread use of highly selected hybrids, especially in developed countries, has led to the progressive extinction of many native breeds in these contexts. Nowadays, safeguarding poultry biodiversity is a key objective in all developed countries, Italy included. As a part of a large cross-sectional national project called ‘Conservation of biodiversity in Italian poultry breeds’, a questionnaire was designed to evaluate the diffusion of native chicken breeds and their relative product markets. The data reveal the poor diffusion of native breeds despite the existence of a niche market for their products. Indeed, increasing consumer concern about conventional production practices and the growing demand for alternative poultry products, which can fetch high retail prices, should be leveraged to encourage the diffusion of native chicken breeds in alternative poultry farming. An important knock-on effect would be the preservation of biodiversity.

**Abstract:**

The intensive use of high-performing strains in poultry production has led to the extinction of several autochthonous chicken breeds and, consequently, loss of genetic variability. Interest in saving biodiversity is growing rapidly and has become a major objective worldwide. The aim of this study was to shed light on the production trends of native Italian poultry breeds and the related market. A questionnaire, which asked about the production cycles, the number of animals and table eggs produced per year and their retail prices was completed by 121 breeders across Italy. The surveyed breeders were divided into two categories: breeders conducting an agrozootechnical farm, referred to as ‘farmers’ (F); and breeders keeping chickens as backyard poultry, referred to as ‘fancy breeders’ (FB). Analysis of the data acquired indicated that animals were mainly slaughtered between 6 and 12 months of age, with F processing more animals per year. The same production trend was observed for table eggs. The recorded retail prices of native chicken products were higher than those for conventional products, but similar to those reported for valuable niche poultry products, such as the Poulet de Bresse in France and organic eggs. Knowledge about these highly valuable markets should be used to encourage the use of local breeds in alternative poultry farming and help protect biodiversity.

## 1. Introduction

According to the Domestic Animal Diversity Information System (DAD-IS), developed and maintained by the Food and Agriculture Organization (FAO), 28% of the 1499 local chicken breeds surveyed worldwide present a conservation status ranking from vulnerable to critically endangered, and 3.4% are already extinct [1]. In Italy, 53 native chicken breeds have been described to date, the majority of which are reported to be endangered or extinct [2]. Fortunately, the last few decades have witnessed significant growth in the interest in native breeds, also arousing the interest of the scientific community, and 22 breeds are currently included in national biodiversity safeguarding project [3].

Local breeds of farm animal have significant socio-cultural and ecological relevance, and their rearing may enhance local communities and reduce the negative impact of intensive farming systems. Over the last century, the overwhelming wide-spread use of just a small number of highly selected breeds has led to the extinction of most native breeds worldwide [4]. Such a situation is greatly emphasised in the poultry sector where only selected strains are reared for meat and egg production, and local breeds have been excluded from intensive productive systems, causing a significant reduction in animal genetic resources and the erosion of many native genotypes [5]. The intense selection practices applied to commercial strains has also led to a reduction in genetic viability, reducing the capacity of these breeds to response to and withstand environmental changes and climate variations.

Over recent years, consumers’ knowledge about climate change and their awareness about the impact that intensive animal production systems may have upon it has increased greatly. Furthermore, problems related to the competition for land and resources have emerged, strengthening consumers’ concern about the sustainability of animal production systems [6]. Increases in consumer knowledge and concern have also been observed in relation to conventional poultry production methods and have led to a raise in the demand for poultry products obtained through alternative farming methods [7,8,9]. Moreover, in developed countries, the rediscovery of local products and traditions and renewed consumer interest in products presenting quality traits that different from those of conventional products have opened the doors to new profitable niche markets. The use of autochthonous chicken breeds to satisfy these new demands of consumers should be encouraged to help safeguard biodiversity, enhancing the diffusion of native chicken breeds by ensuring their use in the production of alternative poultry products. Examples of the economic potentials of poultry genetic resources and alternative farming methods are well reported in France in relation to Label Rouge products and Poulet de Bresse [10], whose production systems require expensive and long rearing conditions that include outdoor access, thus determining the higher retail prices. Despite their higher selling prices compared with those fetched by conventional products, French consumers have shown significant interest in these poultry products obtained from slow growing genotypes [11]. The good reputation of Poulet de Bresse breed in alternative rearing systems has allowed its expansion in other countries, such as Spain [12] and Germany [13], where it is considered a premium product. The inability of the conventional fast-growing hybrids to adapt to rearing in alternative farming systems is well documented for both egg production [14,15] or meat production [16,17,18,19]. From the comparison of conventional hybrids with local breeds reared in free-range or organic conditions, it emerged that the adaptability of native birds to different environmental conditions and their capability to synthetise and transfer nutritional components considered favourable for human health to their tissues was significantly better overall [6]. Poultry products obtained from native pure breeds offer unique features and valuable quality traits that may satisfy the demands of particular consumer segments [11,20,21]. Their meat is generally observed to be darker than that of broilers, and it presents a high protein and low lipid content with a favourable fatty acid profile [11,20,22,23]; eggs from native hens present valuable quality traits, such as a higher yolk to albumen ratio [6,24,25,26,27], higher eggshell breaking strength [6,21] and an optimal fatty acid profile. 

The aim of this study was to evaluate, by means of a national survey, the production of native Italian poultry breeds and their destination markets by evaluating the total units produced per year and the prices at which they are sold.

## 2. Materials and Methods

A questionnaire was designed as a part of a large multi-disciplinary project called ‘Conservation of biodiversity in Italian poultry breeds’ [28] dedicated to the safeguarding, conservation and enhancement of the Italian poultry genetic resources, represented by the many autochthonous breeds that have historically been present in Italy. 

The questionnaire was divided into two parts and the survey included breeders from North (45%), Central (36%), and South (19%) Italian regions. The first part was designed to gather information on breeders themselves, on the chicken and turkey breeds reared and on their housing conditions and management practices. The second part included: local chicken breed productive cycles used by breeders, meat and egg production volumes, products destinations, and relative retail prices. The results of the first part are described and discussed in Castillo et al. [3], whereas the results of the second part are presented and discussed herein.

Of the breeders surveyed, those conducting commercial businesses were included in the farmer category (F). Breeders keeping chickens as backyard poultry were referred to as fancy breeders (FB). F and FB with more than 10 animals of each breed were contacted and invited to participate in the survey; contact details were acquired from a comprehensive list compiled by contacting regional farmer associations and national and local fancy breeder associations. Breeders who agreed to participate were visited by researchers between June 2018 and June 2019, and the questionnaire was filled in by means of face-to-face interviews and farm inspections.

After each farm visit, the survey data were entered into a purpose-built spreadsheet in Microsoft Office Excel [29] using the manual double-entry approach, and subsequently checked for data entry errors. The software JMP 9.0.1 (2009) [30] was used for all statistical analyses. The chi-squared test, followed by Fisher’s test, was used to determine any significant differences in the distribution of the considered items within the two different breeder categories, F and FB, as well as between them. *P*-values less than 0.05 were considered significant. Results are presented as the number and percentage of farmers and fancy breeders for each categorical variable. For certain variables, the sum of the responses obtained from the two breeder categories does not necessarily equal the total number of breeders surveyed, which may reflect the incidence of non-responses or be due to the fact the response to some questions may preclude them from answering.

## 3. Results

**Figure 1:**
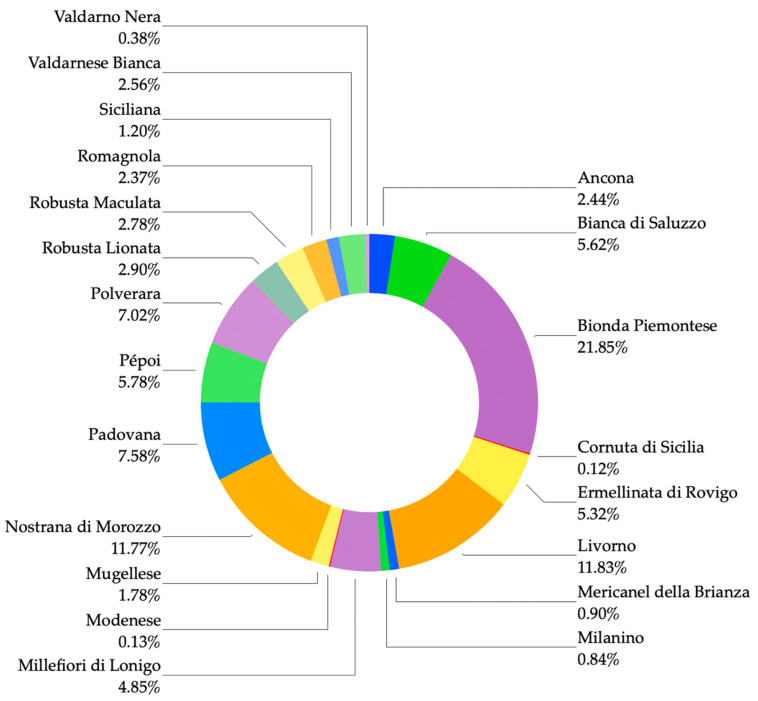
Native Italian poultry breeds recorded during the survey. Phenotypic information are available at: www.pollitaliani.it/en/ accessed on 27 February 2021 [28].

A total of 121 breeders, who agreed to participate in the survey, were visited by researchers; 62% of breeders belong to the F category and the remaining 38% were FB (*p* < 0.01). In total, 21 local chicken breeds for a total of 15,562 individual birds were recorded during the survey (Figure 1; for further information see Castillo et al. [3]).

The type of productive cycle and production area are reported in Table 1. A closed production cycle was used by 61% of all breeders (*p* < 0.01) and by 81% of FB (*p* < 0.01). The F category showed no preference towards either of the two cycle types. Of FB, 19% used an open production cycle, but none of these were rearing birds for the meat production. Half of all F used an open cycle, but relatively few of these were rearing hens for egg production (12%) or for breeding purposes (15%, *p* < 0.05).

When a closed cycle was used by FB, flocks were prevalently being kept as breeders (60%, *p* < 0.01), whereas 20% were destined as laying hens and the remaining 20% were equally distributed between dual-purpose or meat chickens. A difference between the two breeder categories was observed in the closed production system: breeder flocks were predominantly kept by FB (60%, *p* < 0.01), whereas F were more likely to raise flocks for meat production (38%, *p* < 0.05; Table 1).

Considering all breeders, 73% did not collect data on their flocks’ productive performances (*p* < 0.01; Table S1). When this practice was performed, however, data were mainly kept in paper format, rather than digitalised: 21 and 5%, respectively (Table S1). The most frequently recorded data were egg production/day (34%), and live body weight (21%, *p* < 0.05; Table S1). A common practice reported by breeders (63%) was the selling of live birds (*p*<0.01; Table S2)

### 3.1. Meat Production from Autochthonous Chicken Breeds and Destination Markets

Birds were mainly sacrificed and processed within slaughterhouses owned by the breeders themselves (66%, *p* < 0.01; Table 2). The majority of breeders of both categories slaughtered both male and female birds in the 6 to 12 months age range (*p* < 0.01; Table 2). None of the birds slaughtered by FB were aged more than 12 months, whereas 14% of F did sacrifice older birds (*p* < 0.05).

The proportion of breeders rearing birds for meat production with the sole aim of satisfying self-consumption or local markets was similar (45% and 32%, respectively; Table 3). Within the F category, the destination markets for meat products were primarily private citizens only (42%) or a combination of customer categories (37%, including: private citizens, shops, restaurants, and other destinations) (Table 3). Within FB, meat products were mainly produced for the purpose of home consumption (75%, *p* < 0.01).

The number of birds sacrificed by FB was always less than 100 per year (*p* < 0.01) (Table 3). Birds slaughtered by F ranged from less than 100 to more than 500 in a year (Table 3).

The most requested meat product was the partially eviscerated carcass (65%, *p* < 0.01; Table S3). Differences between F and FB were observed in products sold as chicken in pieces and ready-to-cook carcass. FB sold chicken in pieces 32% more often than F (*p* < 0.01), and ready-to-cook carcasses were only sold by FB (12%, *p* < 0.05; Table S3).

The production, rearing system and slaughter procedures of capons were also evaluated. The results are presented in Table S4. A total of 56% of all breeders reared capons (Table S4). The most common method of castration was the surgical bilateral approach (77%, *p* < 0.01), and this procedure was mainly performed on birds aged 30–50 days (*p* < 0.01). All breeders only slaughtered capons aged 6–12 months (Table S4).

### 3.2. Table Egg Production from Autochthonous Chicken Breeds and Destination Markets

Most F kept hens in production for one laying cycle (52%, *p* < 0.01), whereas the proportion of FB keeping hens for one or more cycles was more equally distributed (Table 4). Manual egg collection was performed by almost all breeders (*p* < 0.01). Very few F had an automatised egg collection system (8%; Table 4). The frequency of egg collection was mostly once a day for all breeders (65%, *p* < 0.01). The collection of eggs more than twice a day was also reported in the F category of breeders (3%; Table 4).

Most breeders (64%, *p* < 0.01) both self-consumed and sold the eggs produced. Private citizens were the principal customers (55%, *p* < 0.01), whereas only 15% of breeders exclusively sold eggs to shops and restaurants (Table 5). Breeders producing less than 500 eggs in a year were more common in the FB category, whereas productions of more than 1000 eggs yearly were more often found in the F category (*p* < 0.05; Table 5).

### 3.3. Selling Prices of Meat Products and Table Eggs Obtained from Autochthonous Chicken Breeds

The selling prices of Italian chicken breed products are presented in Table 6. The price of meat products produced by F ranged from less than 10 €/kg to more than 15 €/kg of product. Data on the selling prices of products from FB is largely lacking since only two breeders provided data. 

Most breeders set the selling price of their table eggs at above 0.20 €/egg (*p* < 0.01). Eggs were rarely sold for less than 0.20 €/egg (Table 6).

## 4. Discussion

Italian chicken breeds are receiving increasing amounts of research attention, and studies on their genetics, breeding and productive performances, product quality, rearing management, welfare, and physiological traits have been conducted and published [3]. However, very little data are available about the small-scale production of these breeds and the sale of their products, thus the results of this study contribute to fill this gap. 

The closed productive cycle, mainly present among FB, is a type of productive cycle in which flocks belonging to the same genotype but with different productive purposes are present on the same farm, for example breeders, layers, and meat chicken flocks. In this type of production cycle, the next generation of birds are not sourced externally, but selected from the off-spring of breeders reared on site. Both breeder categories presenting this type of productive cycle reported keeping breeder birds of native chicken breeds, and to produce a number of chicks every year (data not shown). These chicks are then selected as the next generation of breeders or used as hens for table egg production or chickens for meat production. Similar production and rearing systems have been reported in relation to small-scale poultry farming and backyard poultry breeders in several other countries around world [31,32,33]. 

The open production cycle, in which all the animals reared come from external hatcheries or pullet farms, is well known in the context of intensive poultry farming, and in this type of cycle animals are only farmed for the production of fertile eggs, table eggs, or meat. The use of this type of production cycle is a sign of more specialised production [34]. 

The fact that the majority of FB reported to use a closed production cycle and to keep mainly breeders suggests that the production of meat and table eggs is of minor interest and that animals are reared with the prime purpose of maintaining the autochthonous chicken breed strain. The use of an open cycle was mainly observed in F, suggesting that for this category of breeders the raising of chickens is mainly directed towards production. 

The majority of breeders reported to sell live chickens (Table S2). Similar findings were reported by Gondwe and Wollny [31]; according to these authors, in Malawi, Africa, the sale of native breed chickens ranked third as the prime motivation to keep chickens. The surveyed breeders specified that in several cases they sell alive birds to other breeders that will use the purchased animals as breeders. 

Italian chicken breeds are known to be slow growing genotypes and are traditionally dual-purpose breeds, providing both meat and eggs. The reported age at slaughter in Italian breeds is usually 180 days, as, for example, in Milanino [22,35], Pépoi, Padovana, Ermellinata di Rovigo [11], Robusta Maculata [20], Livorno [36], Bionda Piemontese, and Bianca di Saluzzo breeds [37]. In the present study, for both breeder categories and independent of the Italian breed reared, the age at slaughter for both male and female chickens was predominantly between 6 and 12 months of age. In commercial dual-purpose hybrids instead, such as Lohmann Dual and Novogen Dual, the age at slaughter is between 63 and 84 days [38,39,40]. This data confirms that the surveyed local breeds are primarily slow growing genotypes. 

The partially eviscerated carcass, namely, a carcass with the gastro-intestinal tract removed but with the liver and heart still intact, is a traditional product derived from local breeds [22], and in this study it constituted the main meat product obtained from these autochthonous breeds. The data recorded on the destination of poultry meat products shows a prevalence for self-consumption; furthermore, the low number of birds slaughtered per year supports the hypothesis that FB are generally geared towards self-consumption farming. 

In Italy, the capon is a much-appreciated traditional product; its production is a seasonal activity, and the request for capon meat is mainly concentrated during the Christmas period [41]. Capon production is also practiced in some other European countries, such as Poland, Spain, and France, as well as in China, Japan, the USA, and Taiwan [42,43,44]. According to Calick [42], capon production has fortified the breeding of native slow-growing breeds, which may even originate from the local region, examples being the Bresse breed in France and the Castellana Negra in Spain. In Italy, two niche and widely appreciated capons are the Cappone di San Damiani d’Asti and the Cappone di Morozzo, obtained by castrating males belongings to the Bionda Piemontese and Nostrana di Morozzo breeds, respectively. The Cappone di Morozzo has its own label and is included amongst the products of the Slow Food Foundation for Biodiversity [45].

In the present study, capon production was reported by 56% of the farms surveyed, and the surgical procedure was mainly performed between 4 and 7 weeks of age (77%). A similar castration age was also reported for the Sasso-X44 hybrid line (6 weeks of age) [46]. An older castration age (8 weeks) was reported for the Castellana Negra [44], Mos [46], Greenleg Partridge, and Polbar pure breeds [43]. In our study, all breeders slaughtered capons between 6 and 12 months of age. The slaughter ages reported for the Spanish Castellana Negra and Mos breeds (7 and 8.5 months of age, respectively) [44,46] and the Polish Green-legged Partridge and Polbar breeds (6 months of age) also fall within this age range [43].

Native breed hens kept by F for table egg production are mainly reared for a single laying cycle (*p* < 0.01), whereas no preference was noted with regard to the number of cycles among FB. This may reflect a sentimental attachment of FBs towards their flocks, making them more likely to keep the birds for repeated cycles. Similar findings were observed by Elkhoraibi et al. [47]. According to these authors, in the USA, 57% of back-yard chicken owners keep the birds as pets. Another explanation of the lack of preference for one, two or more cycles in this breeder category could be related to aesthetic aspects; indeed, FBs were observed to choose breeds known for their aesthetic qualities, and to keep these chosen birds often for their entire life, despite the deterioration of egg-laying performances overtime. 

The sale of table eggs often occurred hand-in-hand with self-consumption in both breeder categories. From the data collected, we cannot confirm the prime purpose of table egg production; however, the differences observed in the numbers of table eggs sold per year between the two breeder categories strengthen the hypothesis that F mainly produce eggs with the view of selling them, whereas the sale of eggs by FB is more likely related to overproduction.

The low level of mechanization in egg collection revealed in this study confirm egg production to be a small-scale activity for both FB and F. Indeed, considering that the egg and poultry industries are renowned for their high level of mechanization worldwide, the fact that they were not in the contexts analysed here provides support to this conclusion. For example, Scott et al. reported that eggs in large-scale layer poultry farms are mainly collected by automatic conveyer belts, whereas manual egg collection was performed by just 10% of farmers [48].

The breeders reported a high selling price for the meat products and table eggs obtained from native chicken breeds. In fact, 38% of all breeders reported to sell their meat products for 10–15 €/kg, and 42% sold them at prices superior to 15 €/kg. At the time of the close of this survey (June 2019), wholesale selling prices for ready-to-cook chicken carcasses and partially eviscerated carcasses were reported to be 2.33 €/kg and 2.30 €/kg, respectively [49]. The recorded selling prices of native Italian chicken breeds are similar to those reported in France for the Bresse breed, for which partially eviscerated carcasses sell at 13 €/kg. Higher prices are reported for hens and capons whole carcasses of the Bresse breed, 20 €/kg and 25 €/kg, respectively [50].

The price of the meat sold by FB did not exceed 15 €/kg, whose selling prices were equally split between the two lower price ranges, less than 10 €/kg and 10–15 €/kg. Prices lower than 10 €/kg, have also been reported in France for Label Rouge and organic chicken meet [51]. 

The price of table eggs from native chicken breeds reported here are higher than those reported for poultry industries. In the present study, the overall selling prices of table eggs laid by autochthonous hens were mainly 0.20–0.40 €/egg or above 0.40 €/egg (51.51% and 42.42%, respectively). In June 2019, the wholesale price of table eggs was 0.10 €/egg for conventional medium size eggs [52]. Higher selling prices were reported in 2019 for organic eggs sold direct to the public: 0.37 €/egg [53]. Based on these observations, we can affirm that even if table eggs produced by native Italian breeds are not produced according to organic protocols, they can nevertheless fetch equivalent or even higher selling prices.

The results reveal the existence of a small niche market for local chicken breed products in Italy. Pellattiero et al. [23] reported that when consumers choose products, poultry meat products for example, they are generally not aware of their real quality attributes, but instead tend to form expectations based on available clues which, in turn, affect their purchase behaviours and preferences for certain products over others. 

Recently, consumers have also started to become more sensitive to fraudulent claims and the mislabelling of food products, thus product authentication has become a key issue in the food industry [54]. This topic is particularly important when a label or brand is restricted to animals belonging to a single breed; in such cases, the molecular traceability of a product is highly important, providing a means to control the origin of animals, thereby helping to render the supply chain more transparent. In fact, when chicken carcasses from local breeds are marketed, their conformation is different to that of industrial broilers, and the distinction between the two is easy to ascertain. However, distinguishing between the carcasses of local breeds and those obtained from male birds of layer lines, which are more likely to have similar body weights and conformation, would be more difficult, especially when the product is sold in pieces. In those cases, molecular traceability can be a valid support to product authentication [54]. Furthermore, the authentication and molecular traceability of labelled native breed chicken products may enhance consumer confidence and positively modify their purchasing behaviours, encouraging them to favour products from native breeds.

## 5. Conclusions

In the present study, valuable information on the actual rearing and end-use of native Italian chicken breeds in small-scale poultry farms and by ‘back-yard’ fancy breeders in Italy was collected and evaluated. Analysis of the data revealed an overwhelming prevalence of self-consumption poultry farming in FB. The higher numbers of birds slaughtered per year and table eggs produced by F indicate more stable and plentiful production levels. However, the recorded preference for manual egg collection and the tendency of in-house processing animals for meat production, instead of using external slaughterhouses, suggests a still undeveloped level of this kind of poultry farming. The selling prices reported highlight the existence of a unique niche market for these products. 

Knowledge of the existence of a native breeds product niche market and enhanced consumer awareness and demand for alternative products could be used to encourage the rearing of autochthonous breeds by alternative poultry farmers. Finally, enhancing the diffusion of native breeds in alternative farming will help safeguard the genetic resources of Italian poultry.

## Figures and Tables

**Table 1:** Type of production cycle and related production areas: responses from all breeders and divided according to breeder category.

	All Breeders		Farmers		Fancy Breeders		
Variable	*n*	%	*n*	%	*n*	%	χ^2 1^
Type of Cycle:	(*n* = 105)		(*n* = 68)		(*n* = 37)		
Closed Cycle	64 ^A^	60.95	34	50.00	30 ^A^	81.08	**
Open Cycle	41 ^B^	39.05	34	50.00	7 ^B^	18.92	**
Closed Cycle Production Areas:	(*n* = 64)		(*n* = 34)		(*n* = 30)		
Breeders	26 ^A^	40.63	8	23.53	18 ^A^	60.00	**
Meat	16 ^AB^	25.00	13	38.24	3 ^B^	10.00	*
Eggs	11 ^B^	17.19	5	14.71	6 ^B^	20.00	NS
Meet and Eggs	11 ^B^	17.19	8	23.53	3 ^B^	10.00	NS
Open Cycle Production Areas:	(*n* = 41)		(*n* = 34)		(*n* = 7)		
Breeders	8 ^ab^	19.51	5 ^ab^	14.71	3	42.86	NS
Meat	13 ^ab^	31.71	13 ^a^	38.24	0	0.00	*
Eggs	5 ^b^	12.20	4 ^b^	11.76	1	14.29	NS
Meet and Eggs	15 ^a^	36.59	12 ^a^	35.29	3	42.86	NS

^1^ Chi square test for a single variable between the two breeder categories, i.e., within row comparisons; significance levels: ** *p* < 0.01; * *p* < 0.05; NS, non-significant (*p* > 0.05). ^A, B^ Observations with different superscripts within the column are significantly different (χ^2^-test, *p* < 0.01). ^a, b^ Observations with different superscripts within the column are significantly different (χ^2^-test, *p* < 0.05).

**Table 2:** Slaughter information: responses from all breeders and divided according to breeder category.

	All Breeders		Farmers		Fancy Breeders		
Variable	*n*	%	*n*	%	*n*	%	χ^2 1^
Slaughterhouse:	(*n* = 50)		(*n* = 41)		(*n* = 9)		
Internal	33 ^A^	66.00	26 ^a^	63.41	7 ^a^	77.78	NS
External	17 ^B^	34.00	15 ^b^	36.59	2 ^b^	22.22	NS
Male-Age at Slaughter:	(*n* = 36)		(*n* = 28)		(*n* = 8)		
<than 6 months	6 ^B^	16.67	4 ^B^	14.29	2 ^AB^	25.00	NS
Between 6–12 months	26 ^A^	72.22	20 ^A^	71.43	6 ^A^	75.00	NS
˃than 12 months	4 ^B^	11.11	4 ^B^	14.29	0 ^B^	0.00	*
Female-Age at Slaughter:	(*n* = 39)		(*n* = 34)		(*n* = 5)		
<than 6 months	8 ^B^	20.51	6 ^B^	17.65	2	40.00	NS
Between 6–12 months	22 ^A^	56.41	19 ^A^	55.88	3	60.00	NS
˃than 12 months	9 ^B^	23.08	9 ^B^	26.47	0	0.00	NS

^1^ Chi square test for a single variable between the two breeder categories, i.e., within row comparisons; significance levels: * *p* < 0.05; NS, non-significant (*p* > 0.05). ^A, B^ Observations with different superscripts within the column are significantly different (χ^2^-test, *p* < 0.01). ^a, b^ Observations with different superscripts within the column are significantly different (χ^2^-test, *p* < 0.05).

**Table 3:** Destination of Italian chicken breed meat products (self-consumption vs sale) and the distribution between different categories of customers: responses from all breeders and divided according to breeder category.

	All Breeders		Farmers		Fancy Breeders		
Variable	*n*	%	*n*	%	*n*	%	χ^2 1^
Destination of Meat Products:	(*n* = 47)		(*n* = 39)		(*n* = 8)		
Self-consumption	21	44.68	15	38.46	6 ^A^	75.00	NS
Sale	15	31.91	13	33.33	2 ^AB^	25.00	NS
Both	11	23.40	11	28.21	0 ^B^	0.00	NS
Meat Product Customers:	(*n* = 26)		(*n* = 24)		(*n* = 2)		
Shops and restaurants only	3 ^BC^	11.54	3 ^ab^	12.50	0 ^B^	0.00	NS
Private citizens only	10 ^AB^	38.46	10 ^a^	41.67	0 ^B^	0.00	NS
Other destinations only	2 ^C^	7.69	2 ^b^	8.33	0 ^B^	0.00	NS
A combination of the above	11 ^A^	42.31	9 ^a^	37.50	2 ^A^	100.00	NS
Birds Slaughtered/Year (*n*):	(*n* = 36)		(*n* = 29)		(*n* = 7)		
<than 100	16	44.44	9	31.03	7 ^A^	100.00	**
Between 100–500	13	36.11	13	44.83	0 ^B^	0.00	*
˃than 500	7	19.44	7	24.14	0 ^B^	0.00	NS

^1^ Chi square test for a single variable between the two breeder categories, i.e., within row comparisons; significance levels: ** *p* < 0.01; * *p* < 0.05; NS, non-significant (*p* > 0.05). ^A–C^ Observations with different superscripts within the column are significantly different (χ^2^-test, *p* < 0.01). ^a, b^ Observations with different superscripts within the column are significantly different (χ^2^-test, *p* < 0.05).

**Table 4:** Italian chicken breed table egg production characteristics: responses from all breeders and divided according to breeder category.

	All Breeders		Farmers		Fancy Breeders		
Variable	*n*	%	*n*	%	*n*	%	χ^2 1^
Cycles of Egg Production:	(*n* = 76)		(*n* = 42)		(*n* = 34)		
1 laying cycle	33	43.42	22 ^A^	52.38	11	32.35	NS
2 laying cycle	21	27.63	12 ^B^	28.57	9	26.47	NS
˃than 2 laying cycles	22	28.95	8 ^B^	19.05	14	41.18	NS
System of Egg Collection:	(*n* = 60)		(*n* = 39)		(*n* = 21)		
Manual	57 ^A^	95.00	36 ^A^	92.31	21 ^A^	100.00	NS
Mechanical	3 ^B^	5.00	3 ^B^	7.69	0 ^B^	0.00	NS
Frequency of Egg Collection:	(*n* = 57)		(*n* = 38)		(*n* = 19)		
Once a day	37 ^A^	64.91	22 ^A^	57.89	15 ^A^	78.95	NS
Twice a day	19 ^B^	33.33	15 ^A^	39.47	4 ^B^	21.05	NS
˃than twice a day	1 ^C^	1.75	1 ^B^	2.63	0 ^B^	0.00	NS

^1^ Chi square test for a single variable between the two breeder categories, i.e., within row comparisons; significance levels: NS, non-significant (*p* > 0.05). ^A–C^ Observations with different superscripts within the column are significantly different (χ^2^-test, *p* < 0.01).

**Table 5:** Destination markets of Italian chicken breed table eggs: responses from all breeders and divided according to breeder category.

	All Breeders		Farmers		Fancy Breeders		
Variable	*n*	%	*n*	%	*n*	%	χ^2 1^
Destination of Table Eggs	(*n* = 42)		(*n* = 29)		(*n* = 13)		
Self-consumption	9 ^B^	21.43	6 ^B^	20.69	3 ^ab^	23.08	NS
Sale to customers	6 ^B^	14.29	4 ^B^	13.79	2 ^b^	15.38	NS
Both	27 ^A^	64.29	19 ^A^	65.52	8 ^a^	61.54	NS
Table Egg Customers:	(*n* = 33)		(*n* = 23)		(*n* = 10)		
Shops and restaurants only	5 ^B^	15.15	4 ^B^	17.39	1 ^b^	10.00	NS
Private citizens only	18 ^A^	54.55	12 ^A^	52.17	6 ^a^	60.00	NS
Other destinations	3 ^B^	9.09	2 ^B^	8.70	1 ^b^	10.00	NS
A combination of the above	7 ^B^	21.21	5 ^AB^	21.74	2 ^b^	20.00	NS
Table Eggs Sold/Year (*n*):	(*n* = 38)		(*n* = 25)		(*n* = 13)		
<than 500	7	18.42	2 ^B^	8.00	5	38.46	*
Between 500–1000	16	42.11	10 ^A^	40.00	6	46.15	NS
˃than 1000	15	39.47	13 ^A^	52.00	2	15.38	*

^1^ Chi square test for a single variable between the two breeder categories, i.e., within row comparisons; significance levels: * *p* < 0.05; NS, non-significant (*p* > 0.05). ^A, B^ Observations with different superscripts within the column are significantly different (χ^2^-test, *p* < 0.01). ^a, b^ Observations with different superscripts within the column are significantly different (χ^2^-test, *p* < 0.05).

**Table 6:** Prices of meat products and table eggs obtained from Italian chicken breeds: responses from all breeders and divided according to breeder category.

	All Breeders		Farmers		Fancy Breeders		
Variable	*n*	%	*n*	%	*n*	%	χ^2 1^
Meat Product Price	(*n* = 26)		(*n* = 24)		(*n* = 2)		
<than 10 €/kg	5	19.23	4	16.67	1	50.00	NS
Between 10–15 €/kg	10	38.46	9	37.50	1	50.00	NS
˃than 15 €/kg	11	42.31	11	45.83	0	0.00	NS
Table Egg Unit Price	(*n* = 33)		(*n* = 23)		(*n* = 10)		
<than 0.20 €/egg	2 ^B^	6.07	1 ^B^	4.34	1	10.00	NS
Between 0.20–0.40 €/egg	17 ^A^	51.51	11 ^A^	47.83	6	60.00	NS
˃than 0.40 €/egg	14 ^A^	42.42	11 ^A^	47.83	3	30.00	NS

^1^ Chi square test for a single variable between the two breeder categories, i.e., within row comparisons; significance levels: NS, non-significant (*p* > 0.05). ^A, B^ Observations with different superscripts within the column are significantly different (χ^2^-test, *p* < 0.01).

## Data Availability

The data presented in this study are available on request from the corresponding author.

## Supplementary Materials

The following are available online at https://www.mdpi.com/2076-2615/11/3/629/s1, Table S1: Productive performance data collection: responses from all breeders and divided according to breeder category, Table S2: Sale of alive birds: responses from all breeders and divided according to breeder category, Table S3: Italian poultry breeds meat products produced: responses from all breeders and divided according to breeder category, Table S4: Italian breeds capons production, rearing and slaughtering: responses from all breeders and divided according to breeder category.

## References

[1] Food and Agriculture Organization of the United Nations. http://www.fao.org/dad-is/risk-status-of-animal-genetic-resources/en/.

[2] Zanon A., Alberto S. (2001). Identificazione e salvaguardia genetica delle razze avicole italiane. Ann. Fac. Med. Vet. Parma.

[3] Castillo A., Gariglio M., Franzoni A., Soglia D., Sartore S., Buccioni A., Mannelli F., Cassandro M., Cendron F., Castellini C. (2021). Overview of Native Chicken Breeds in Italy: Conservation Status and Rearing Systems in Use. Animal.

[4] Directorate-General for Agriculture and Rural Development (2020). Preparatory Action EU Plant and Animal Genetic Resources—Executive Summary.

[5] Cendron F., Perini F., Mastrangelo S., Tolone M., Criscione A., Bordonaro S., Iaffaldano N., Castellini C., Marzoni M., Buccioni A. (2020). Genome-Wide SNP Analysis Reveals the Population Structure and the Conservation Status of 23 Italian Chicken Breeds. Animal.

[6] Di Rosa A.R., Chiofalo B., Presti V.L., Chiofalo V., Liotta L. (2020). Egg Quality from Siciliana and Livorno Italian Autochthonous Chicken Breeds Reared in Organic System. Animal.

[7] Castellini C., Dal Bosco A., Petracci M., Berri C. (2017). Animal welfare and poultry meat in alternative production systems (and ethics of poultry meat production). Poultry quality evaluation. Quality attributes and Consumers Value.

[8] Lordelo M., Cid J., Cordovil C.M., Alves S.P., Bessa R.J., Carolino I. (2020). A comparison between the quality of eggs from in-digenous chicken breeds and that from commercial layers. Poult. Sci..

[9] Tasoniero G., Cullere M., Baldan G., Zotte A.D. (2018). Productive performances and carcase quality of male and female Italian Padovana and Polverara slow-growing chicken breeds. Ital. J. Anim. Sci..

[10] Baéza E., Chartrin P., Le Bihan-Duval E., Lessire M., Besnard J., Berri C. (2009). Does the chicken genotype ‘Géline de Touraine’ have specific carcass and meat characteristics?. Animal.

[11] Zanetti E., De Marchi M., Dalvit C., Molette C., Remignon H., Cassandro M. (2010). Carcase characteristics and qualitative meat traits of three Italian local chicken breeds. Br. Poult. Sci..

[12] Torres A., Muth P.C., Capote J., Rodríguez C., Fresno M., Zárate A.V. (2019). Suitability of dual-purpose cockerels of 3 different genetic origins for fattening under free-range conditions. Poult. Sci..

[13] Muth P.C., Ghaziani S., Klaiber I., Zárate A.V. (2018). Are carcass and meat quality of male dual-purpose chickens competitive compared to slow-growing broilers reared under a welfare-enhanced organic system?. Org. Agric..

[14] Leenstra F., Maurer V., Bestman M., Van Sambeek F., Zeltner E., Reuvekamp B., Galea F., Van Niekerk T. (2012). Performance of commercial laying hen genotypes on free range and organic farms in Switzerland, France and The Netherlands. Br. Poult. Sci..

[15] Küçükyılmaz K., Bozkurt M., Herken E.N., Çınar M., Çatlı A.U., Bintaş E., Çöven F. (2012). Effects of Rearing Systems on Performance, Egg Characteristics and Immune Response in Two Layer Hen Genotype. Asian-Australas. J. Anim. Sci..

[16] Branciari R., Mugnai C., Mammoli R., Miraglia D., Ranucci D., Bosco A.D., Castellini C. (2009). Effect of genotype and rearing system on chicken behavior and muscle fiber characteristics1. J. Anim. Sci..

[17] Castellini C., Mugnai C., Pedrazzoli M., Dal Bosco A. Productive performance and carcass traits of Leghorn chickens and their crosses reared according to the organic farming system. Proceedings of the Atti XII European Poultry Conference.

[18] Castellini C., Bosco A.D., Mugnai C., Bernardini M. (2002). Performance and behaviour of chickens with different growing rate reared according to the organic system. Ital. J. Anim. Sci..

[19] Castellini C., Mugnai C., Dal Bosco A. (2002). Meat quality of three chicken genotypes reared according to the organic system. Ital. J. Food Sci..

[20] Rizzi C., Marangon A., Chiericato G.M. (2007). Effect of Genotype on Slaughtering Performance and Meat Physical and Sensory Characteristics of Organic Laying Hens. Poult. Sci..

[21] Mugnai C., Bosco A.D., Castellini C. (2009). Effect of rearing system and season on the performance and egg characteristics of Ancona laying hens. Ital. J. Anim. Sci..

[22] Mosca F., Zaniboni L., Stella S., Kuster C., Iaffaldano N., Cerolini S. (2018). Slaughter performance and meat quality of Milanino chickens reared according to a specific free-range program. Poult. Sci..

[23] Pellattiero E., Tasoniero G., Cullere M., Gleeson E., Baldan G., Contiero B., Zotte A.D. (2020). Are Meat Quality Traits and Sensory Attributes in Favor of Slow-Growing Chickens?. Animal.

[24] Rizzi C., Marangon A. (2012). Quality of organic eggs of hybrid and Italian breed hens. Poult. Sci..

[25] Sirri F., Zampiga M., Soglia F., Meluzzi A., Cavani C., Petracci M. (2018). Quality characterization of eggs from Romagnola hens, an Italian local breed. Poult. Sci..

[26] Rizzi C., Chiericato G.M. (2005). Organic farming production. Effect of age on the productive yield and egg quality of hens of two commercial hybrid lines and two local breeds. Ital. J. Anim. Sci..

[27] Zanon A., Beretti V., Superchi P., Zambini E.M., Sabbioni A. (2006). Physico-chemical characteristics of eggs from two Italian autochthonous chicken breeds: Modenese and Romagnolo. World’s Poult. Sci. J..

[28] Conservation of Biodiversity in Italian Poultry Breeds (TuBAvI). https://www.pollitaliani.it/en/.

[29] (2019). Microsoft Excel.

[30] (2009). 30. JMP, 9.0.1.

[31] Gondwe T.N., Wollny C.B.A. (2007). Local chicken production system in Malawi: Household flock structure, dynamics, management and health. Trop. Anim. Health Prod..

[32] Abdelqader A., Wollny C.B.A., Gauly M. (2007). Characterization of local chicken production systems and their potential under different levels of management practice in Jordan. Trop. Anim. Health Prod..

[33] Khan A. (2008). Indigenous breeds, crossbreds and synthetic hybrids with modified genetic and economic profiles for rural family and small scale poultry farming in India. World’s Poult. Sci. J..

[34] Food and Agricultural Organization of the United Nations. http://www.fao.org/3/Y4628E/y4628e03.htm.

[35] Mosca F., Madeddu M., Mangiagalli M.G., Colombo E., Cozzi M.C., Zaniboni L., Cerolini S. (2015). Bird density, stress markers and growth performance in the Italian chicken breed Milanino. J. Appl. Poult. Res..

[36] Marzoni M., Castillo A., Franzoni A., Nery J., Fortina R., Romboli I., Schiavone A. (2020). Effects of Dietary Quebracho Tannin on Performance Traits and Parasite Load in an Italian Slow-Growing Chicken (White Livorno Breed). Animal.

[37] Soglia D., Sartore S., Maione S., Schiavone A., Dabbou S., Nery J., Zaniboni L., Marelli S., Sacchi P., Rasero R. (2020). Growth Performance Analysis of Two Italian Slow-Growing Chicken Breeds: Bianca di Saluzzo and Bionda Piemontese. Animal.

[38] Siekmann L., Meier-Dinkel L., Janisch S., Altmann B., Kaltwasser C., Sürie C., Krischek C. (2018). Carcass Quality, Meat Quality and Sensory Properties of the Dual-Purpose Chicken Lohmann Dual. Foods.

[39] Mueller S., Kreuzer M., Siegrist M., Mannale K., Messikommer R., Gangnat I.D.M. (2018). Carcass and meat quality of dual-purpose chickens (Lohmann Dual, Belgian Malines, Schweizerhuhn) in comparison to broiler and layer chicken types. Poult. Sci..

[40] Mueller S., Taddei L., Albiker D., Kreuzer M., Siegrist M., Messikommer R., Gangnat I. (2020). Growth, carcass, and meat quality of 2 dual-purpose chickens and a layer hybrid grown for 67 or 84 D compared with slow-growing broilers. J. Appl. Poult. Res..

[41] Sirri F., Bianchi M., Petracci M., Meluzzi A. (2009). Influence of partial and complete caponization on chicken meat quality. Poult. Sci..

[42] Calik J. (2014). Capon Production–Breeding Stock, Rooster Castration And Rearing Methods, And Meat Quality—A Review. Ann. Anim. Sci..

[43] Kwiecień M., Kasperek K., Tomaszewska E., Muszyński S., Jeżewska-Witkowska G., Winiarska-Mieczan A., Grela E., Kamińska E. (2018). Effect of Breed and Caponisation on the Growth Performance, Carcass Composition, and Fatty Acid Profile in the Muscles of Greenleg Partridge and Polbar Breeds. Braz. J. Poult. Sci..

[44] Miguel J., Ciria J., Asenjo B., Calvo J. (2008). Effect of caponisation on growth and on carcass and meat characteristics in Castellana Negra native Spanish chickens. Animal.

[45] Slow Food Foundation for Biodiversity Morozzo Capon. https://www.fondazioneslowfood.com/it/presidi-slow-food/cappone-di-morozzo/.

[46] Franco D., Pateiro M., Rois D., Vázquez J.A., Lorenzo J.M., Rodriguez J.M.L. (2016). Effects of Caponization on Growth Performance, Carcass and Meat Quality of Mos Breed Capons Reared in Free-Range Production System. Ann. Anim. Sci..

[47] Elkhoraibi C., Blatchford R.A., Pitesky M.E., Mench J.A. (2014). Backyard chickens in the United States: A survey of flock owners. Poult. Sci..

[48] Scott A.B., Singh M., Toribio J.-A., Hernández-Jover M., Barnes B., Glass K., Moloney B., Lee A., Groves P. (2017). Comparisons of management practices and farm design on Australian commercial layer and meat chicken farms: Cage, barn and free range. PLoS ONE.

[49] ISMEA Mercati. http://www.ismeamercati.it/flex/cm/pages/ServeBLOB.php/L/IT/IDPagina/796#MenuV.

[50] Volaille Fines. http://volaillesfines.com/boutique-en-ligne/.

[51] Réseau des Nouvelles des Marchés. https://rnm.franceagrimer.fr/prix?M2501:12MOIS.

[52] ISMEA Mercati. http://www.ismeamercati.it/flex/cm/pages/ServeBLOB.php/L/IT/IDPagina/792#MenuV.

[53] ISMEA Mercati. http://www.ismeamercati.it/flex/cm/pages/ServeBLOB.php/L/IT/IDPagina/10545.

[54] Soglia D., Sacchi P., Sartore S., Maione S., Schiavone A., De Marco M., Bottero M.T., Dalmasso A., Pattono D., Rasero R. (2017). Distinguishing industrial meat from that of indigenous chickens with molecular markers. Poult. Sci..

