# Supplementary material for: Overview of Native Chicken Breeds in Italy: Small Scale Production and Marketing
Source: Animals (Basel). 2021 Feb 27;11(3):629. doi: 10.3390/ani11030629 (PMC7996916; doi:10.3390/ani11030629)
Supplement: Supplementary file 1 [file animals-11-00629-s001.pdf]

animals

MDPI

Article

Overview of Native Chicken Breeds in Italy: Small Scale Production and Marketing

Alessandro Franzoni [1], Marta Gariglio [1], Annelisse Castillo [1], Dominga Soglia [1], Stefano Sartore [1], Arianna Buccioni [2], Federica Mannelli [2], Martino Cassandro [3], Filippo Cendron [3], Cesare Castellini [4], Alice Cartoni Mancinelli [4], Silvia Cerolini [5], Ahmad Abdel Sayed [5], Nicolaia Iaffaldano [6], Michele Di Iorio [6], Margherita Marzoni [7], Sonia Salvucci [7] and Achille Schiavone [1,*]

[1] Dipartimento di Scienze Veterinarie, Università degli Studi di Torino, Largo Paolo Braccini 2, 10095 Grugliasco, Italy; alessandro.franzoni@unito.it (A.F.); marta.gariglio@unito.it (M.G.); annelisse.castillogarrido@unito.it (A.C.); dominga.soglia@unito.it (D.S.); stefano.sartore@unito.it (S.S.);
[2] Dipartimento di Scienze e Tecnologie Agrarie, Alimentari, Ambientali e Forestali, Università di Firenze, Via delle Cascine 5, 50144 Firenze, Italy; arianna.buccioni@unifi.it (A.B.); federica.mannelli@unifi.it (F.M.)
[3] Department of Agronomy, Food, Natural Resources, Animals and Environment (DAFNAE), Università di Padova, Viale dell'Università 16, 35020 Legnaro (PD), Italy; martino.cassandro@unipd.it (M.C.); filippo.cendron@phd.unipd.it (F.C.)
[4] Dipartimento di Scienze Agrarie, Alimentari e Ambientali, Università di Perugia, Borgo XX Giugno 74, 06121 Perugia, Italy; cesare.castellini@unipg.it (C.C.); alice.cartonimancinelli@unipg.it (A.C.M.)
[5] Dipartimento di Medicina Veterinaria, Università degli Studi di Milano, Via dell'Università 6, 26900 Lodi, Italy; silvia.cerolini@unimi.it (S.C.); ahmad.abdel@unimi.it (A.A.S.)
[6] Dipartimento Agricoltura, Ambiente e Alimenti, Università degli Studi del Molise, Via Francesco De Sanctis, Campobasso, Italy; nicolaia@unimol.it (N.I.); michele.diiorio@unimol.it (M.D.I.)
[7] Dipartimento di Scienze Veterinarie, Università di Pisa, Viale delle Piagge 2, 56124 Pisa, Italy; margherita.marzoni@unipi.it (M.M.); sonia.salvucci@unipi.it (S.S.)
* Correspondence: achille.schiavone@unito.it; Tel.: +39-011-6709208

Supplementary Materials: Table S1: Productive performance data collection: responses from all breeders and divided according to breeder category, Table S2: Sale of alive birds: responses from all breeders and divided according to breeder category, Table S3: Italian poultry breeds meat products produced: responses from all breeders and divided according to breeder category, Table S4: Italian breeds capons production, rearing and slaughtering: responses from all breeders and divided according to breeder category.

Table S1. Productive performance data collection: responses from all breeders and divided according to breeder category.

	All Breeders		Farmers		Fancy Breeders		
Variable	*n*	%	*n*	%	*n*	%	χ^2 [1]
Productive Performance Data Collection	(*n* = 85)		(*n* = 62)		(*n* = 23)		
Yes. Digital	4 ^{C}	4.71	3 ^{C}	4.84	1 ^{BC}	4.35	NS
Yes. Paper	18 ^{B}	21.18	12 ^{B}	19.35	6 ^{B}	26.09	NS
Yes. Digital & Paper	1 ^{C}	1.18	1 ^{C}	1.61	0 ^{C}	0.00	NS
No	62 ^{A}	72.94	46 ^{A}	74.19	16 ^{A}	69.57	NS

Collected Data	(*n* = 56)		(*n* = 39)		(*n* = 17)		
Live Body weight	12 [ab]	21.43	9 [ab]	23.08	3	17.65	NS
Feed Consumption	9 [b]	16.07	6 [ab]	15.38	3	17.65	NS
Eggs Production/ Day	19 [a]	33.93	14 [a]	35.90	5	29.41	NS
Egg weight	9 [b]	16.07	5 [b]	12.82	4	23.53	NS
Others	7 [b]	12.50	5 [b]	12.82	2	11.76	NS

[1] Chi square test for a single variable between the two breeder categories, i.e. within row comparisons; significance levels: NS, non-significant ($p > 0.05$). [A–C] Observations with different superscripts within the column are significantly different (χ^2-test $p < 0.01$). [a, b] Observations with different superscripts within the column are significantly different (χ^2-test, $p < 0.05$).

Table 2. Sale of alive birds: responses from all breeders and divided according to breeder category.

	All Breeders		Farmers		Fancy Breeders		
**Variable**	***n***	**%**	***n***	**%**	***n***	**%**	χ^2 [1]
Sale of Alive Birds	(*n* = 113)		(*n* = 73)		(*n* = 40)		
Yes	71 [A]	62.83	44 [a]	60.27	27 [A]	67.50	NS
No	42 [B]	37.17	29 [b]	39.73	13 [B]	32.50	NS

[1] Chi square test for a single variable between the two breeder categories, i.e. within row comparisons; significance levels: NS, non-significant ($p > 0.05$). [A, B] Observations with different superscripts within the column are significantly different (χ^2-test $p < 0.01$). [a, b] Observations with different superscripts within the column are significantly different (χ^2-test, $p < 0.05$).

Table S3. Italian poultry breeds meat products produced: responses from all breeders and divided according to breeder category.

	All Breeders		Farmers		Fancy Breeders		
**Variable**	***n***	**%**	***n***	**%**	***n***	**%**	χ^2 [1]
Meat product types	(n = 46)		(n = 38)		(n = 8)		
Partially eviscerated carcass	30 [A]	65.22	27 [A]	71.05	3	37.50	NS
Ready-to-cook carcass	1 [C]	2.17	0 [C]	0.00	1	12.50	*
Pieces	5 [BC]	10.87	2 [BC]	5.26	3	37.50	**
Processed meat	2 [BC]	4.35	2 [BC]	5.26	0	0.00	NS
Combinations of the above solutions	8 [B]	17.39	7 [AB]	18.42	1	12.50	NS

[1] Chi square test for a single variable between the two breeder categories, i.e. within row comparisons; significance levels: ** $p < 0.01$; * $p < 0.05$; NS, non-significant ($p > 0.05$). [A–C] Observations with different superscripts within the column are significantly different (χ^2-test $p < 0.01$).

Table S4. Italian breeds capons production, rearing and slaughtering: responses from all breeders and divided according to breeder category.

	**All Breeders**		**Farmers**		**Fancy Breeders**		
**Variable**	***n***	**%**	***n***	**%**	***n***	**%**	χ^{2} [1]
Cappons	(*n* = 55)		(*n* = 46)		(*n* = 9)		
Yes	31	56.36	26	56.52	5	55.56	NS
No	24	43.64	20	43.48	4	44.44	NS
Castration method	(*n* = 31)		(*n*=26)		(*n*=5)		
Surgical Unilateral	7 ^{B}	22.58	6^{B}	23.08	1^{B}	20.00	NS
Surgical Bilateral	24 ^{A}	77.42	20^{A}	76.92	4^{A}	80.00	NS
Age at Castration	(*n* = 29)		(*n* = 24)		(*n* = 5)		
< than 30 days old	2 ^{B}	6.90	1 ^{B}	4.17	1	20.00	NS
Between 30 - 50 days old	22 ^{A}	75.86	19 ^{A}	79.17	3	60.00	NS
> than 50 days old	5 ^{B}	17.24	4 ^{B}	16.67	1	20.00	NS
Capons Age at Slaughter	(*n* = 29)		(*n* = 25)		(*n* = 4)		
< than 6 months old	0 ^{B}	0.00	0 ^{B}	0.00	0 ^{B}	0.00	-
Between 6 - 12 months old	29 ^{A}	100.00	25 ^{A}	100.00	4 ^{A}	100.00	NS
> than 12 months old	0 ^{B}	0.00	0 ^{B}	0.00	0 ^{B}	0.00	-

[1] Chi square test for a single variable between the two breeder categories, i.e. within row comparisons; significance levels: NS, non-significant ($p > 0.05$). $^{A, B}$ Observations with different superscripts within the column are significantly different (χ^2-test, $p < 0.01$).
